# In situ Formation of Polymer Microparticles in Bacterial Nanocellulose Using Alternative and Sustainable Solvents to Incorporate Lipophilic Drugs

**DOI:** 10.3390/pharmaceutics15020559

**Published:** 2023-02-07

**Authors:** Tom Bellmann, Jana Thamm, Uwe Beekmann, Dana Kralisch, Dagmar Fischer

**Affiliations:** 1 Division of Pharmaceutical Technology and Biopharmacy, Friedrich-Alexander-Universität Erlangen-Nürnberg, Cauerstraße 4, 91058 Erlangen, Germany; 2 Pharmaceutical Technology and Biopharmacy, Friedrich-Schiller-University Jena, Lessingstraße 8, 07743 Jena, Germany; 3 JeNaCell GmbH—An Evonik Company, Göschwitzer Straße 22, 07745 Jena, Germany; 4 Evonik Industries AG, Rellinghauser Straße 1-11, 45128 Essen, Germany

**Keywords:** bacterial nanocellulose, microparticles, PLGA, drug delivery, cannabidiol, boswellic acids

## Abstract

Bacterial nanocellulose has been widely investigated in drug delivery, but the incorporation of lipophilic drugs and controlling release kinetics still remain a challenge. The inclusion of polymer particles to encapsulate drugs could address both problems but is reported sparely. In the present study, a formulation approach based on in situ precipitation of poly(lactic-co-glycolic acid) within bacterial nanocellulose was developed using and comparing the conventional solvent *N*-methyl-2-pyrrolidone and the alternative solvents poly(ethylene glycol), Cyrene^TM^ and ethyl lactate. Using the best-performing solvents *N*-methyl-2-pyrrolidone and ethyl lactate, their fast diffusion during phase inversion led to the formation of homogenously distributed polymer microparticles with average diameters between 2.0 and 6.6 µm within the cellulose matrix. Despite polymer inclusion, the water absorption value of the material still remained at ~50% of the original value and the material was able to release 32 g/100 cm^2^ of the bound water. Mechanical characteristics were not impaired compared to the native material. The process was suitable for encapsulating the highly lipophilic drugs cannabidiol and 3-O-acetyl-11-keto-β-boswellic acid and enabled their sustained release with zero order kinetics over up to 10 days. Conclusively, controlled drug release for highly lipophilic compounds within bacterial nanocellulose could be achieved using sustainable solvents for preparation.

## 1. Introduction

Bacterial nanocellulose (BNC) is a renewable biopolymer synthesized by various acetic acid bacteria, e.g., *Komagataeibacter xylinus*. Its unique features including high purity, mechanical and thermal stability, biocompatibility, and exceptional water binding characteristics predestinate the material for drug delivery applications and inspired plentiful investigations in this field [1,2]. However, the majority (~80%) of the publications describe the incorporation of highly water-soluble drugs and the few studies aimed to overcome the hydrophilicity of BNC describe either the solubility improvement of the respective drug, the incorporation of disperse systems or the chemical modification of the nanocellulose fibrils by the coupling of lipophilic moieties [3].

A highly desirable formulation approach is the incorporation of polymeric matrices within the BNC network to create lipophilic compartments for poorly water-soluble drugs and, at the same time, enable a modification of the drug release due to the comparatively low diffusivity of the incorporated drugs within the polymer matrix. Up to now, the addition of polymeric matrices into BNC in the context of drug delivery has been investigated only in a small number of publications. Examples include the loading of BNC with polymer nanoparticles, e.g., poly(ethylene oxide)-b-poly(caprolactone) [4], nanochitosan dots [5], zein [6] or hyperbranched polycationic polysaccharides [7] as well as the replacement of the cellulose bound water with solutions of polymers, e.g., poloxamers [8]. All these publications describe the incorporation of particles with diameters below 200 nm using an absorption technique. However, when aiming for controlled-release applications, microparticles are preferentially applied rather than nanoparticles to realize sustained release profiles since the prolonged diffusional pathways translate to extended-release times [9]. However, due to their large diameter a homogenous post-synthesis incorporation of pre-formulated microparticles into BNC via absorption techniques is not feasible, since large fractions of micron-sized particles are immobile within the dense fibril network [10].

Poly(lactic-*co*-glycolic acid) (PLGA) is a popular polymer for the formation of microparticles for drug delivery due to its biodegradability, biocompatibility, and tunable features such as degradation time or hydrophobicity. Another major advantage compared to other medically used polymers is that the starting materials for the polymer synthesis, lactic acid, and glycolic acid, can be produced by renewable methods based on fermentation [11]. The formulation of PLGA microparticles can be realized by a variety of methods including emulsion solvent evaporation [12] or diffusion [13], coacervation [14], spray drying [15], microfluidics [16], and many other approaches [17]. Of these methods, spray drying is the most commonly, also commercially, used manufacturing method due to its reproducibility, scalability, and cost-effectiveness [18,19]. Microfluidic devices are currently gaining attention due to the mild processing conditions, scalability, and precise control over the resulting particle size and shape [20]. Phase inversion from supercritical fluids favors the advantage of the rapid formation of polymer membranes and particles and the complete extraction of the applied solvent [21,22]. However, these processes are more difficult to scale up and require high initial investments for the manufacturing process [23].

All particle manufacturing methods have in common that PLGA is dissolved in an organic solvent followed by dispersion in an external phase of a non-solvent (usually water) before the solubility conditions are changed so that the polymer precipitates as particles, entrapping dissolved or dispersed drugs. In most cases, the change in solubility conditions occurs by removal of the organic solvent, for example, by evaporation or diffusion into the aqueous phase [17]. However, a major drawback of these techniques is the use of toxic and environmentally harmful solvents for the dissolution of the polymer. For example, the frequently used solvent *N*-methyl-2-pyrrolidone (NMP) is classified as a “solvent to be limited” with a permissible daily exposure of 5.3 mg/day by the International Council for Harmonisation of Technical Requirements for Pharmaceuticals for Human Use Quality guideline Q3C(R8) [24]. Accordingly, the interest of researchers and pharmaceutical manufacturers in replacing these solvents with non-toxic, eco-friendly alternatives is growing rapidly [25]. These alternatives are usually labeled “alternative”, “green” or “sustainable”. The term “alternative” describes solvents such as poly(ethylene glycol) (PEG), which can replace potentially harmful solvents due to their non-toxicity, recyclability, and low flammability but are neither obtained from renewable resources nor biodegradable [26]. PEG with a molar mass of 400 g mol^−1^ can be categorized as a safe solvent, since its molecular weight is high enough not to be oxidized to toxic metabolites in humans and low enough for renal secretion, ensuring its clearance from the body [27]. After oral application LD_50_ values above 14,000 mg/kg are reported [28]. Therefore, it is FDA-approved for drug applications including parenteral use [29]. “Green” solvents have better environmental, health, and safety properties than their conventional counterparts considering aspects such as biodegradability or ozone depletion [30]. “Sustainable” solvents have properties similar to green solvents but are additionally obtained from renewable sources without the use of petrochemicals [31]. Examples of green and sustainable solvents are Cyrene^TM^ (dihydrolevoglucosenone, Cyr), which is synthesized from cellulose [32], and ethyl lactate (EL), which can be obtained from corn [33]. Toxicologically, Cyr demonstrated LD_50_ values above 2000 mg/kg without mutagenicity indicating its safe use [34]. Additionally, for ethyl lactate, LD_50_ values above 2000 mg/kg were reported. Ethyl lactate is rapidly enzymatically hydrolyzed to ethanol and lactic acid, which both can be metabolized in the body [35]. Due to its biocompatibility ethyl lactate is approved by WHO as a food additive [36].

In the present study, an incorporation strategy for lipophilic drugs into BNC based on the in situ phase inversion of PLGA solutions directly within the aqueous cellulose network has been investigated. The conventional solvent NMP and the alternatives PEG with 400 g mol^−1^, Cyr, and EL have been used for the dissolution, loading, and phase inversion of PLGA. The main goals were to realize a homogenous incorporation of drug-containing polymer microparticles within BNC, minimize the influence of the process on the water binding and mechanical characteristics of the material, the reduction of the environmental impact of the proposed method, and the realization of a prolonged release for the incorporated drugs. The best-performing incorporation approaches were investigated regarding their drug delivery capabilities by the incorporation and loading of two different natural, highly lipophilic drugs, the antimicrobial cannabidiol (CBD) from *Cannabis sativa* [37] as well as the anti-inflammatory 3-O-acetyl-11-keto-β-boswellic acid (AKBA) from *Boswellia serrata* [38].

## 2. Materials and Methods

### 2.1. Preparation of BNC Fleeces

The synthesis of BNC was carried out on an up-scaled pilot plant as described by Beekmann et al. [39]. Briefly, BNC fleeces were obtained by static cultivation of *Komagataeibacter xylinus* (DSM 14666, German Collection of Microorganisms and Cell Cultures DSMZ, Germany) in Hestrin-Schramm medium in a 1 m^2^ pilot plant at 28 °C for seven days. After synthesis, fleeces were boiled in 0.1 M sodium hydroxide solution (Carl Roth GmbH and Co. KG, Karlsruhe, Germany) and washed with deionized water until neutral pH for purification. Circular BNC fleeces with a 15 mm diameter were punched out of the BNC layer and finally sterilized by autoclaving (121 °C, 20 min, 2 bar). The mass and dimensions of BNC fleeces were characterized to calculate surface area and volume according to Müller et al. [40].

### 2.2. In Situ Formulation of PLGA Particles in BNC Fleeces

Loading solutions were obtained by dissolving PLGA (Resomer^®^ RG502, lactide:glycolide ratio 50:50, 7000–17,000 g mol^−1^, ester terminated, Evonik Industries AG, Essen, Germany) in either NMP, PEG 400 (both Carl Roth), Cyr or EL (both Merck KGaA, Darmstadt, Germany) in concentrations of 10 or 20 mg mL^−1^. Sudan III (Merck) was added to the solutions at a concentration of 0.05 mg mL^−1^ as a lipophilic model compound to evaluate drug distribution. BNC was loaded by immersion of the fleeces in 10.0 mL polymer solution under orbital shaking (IKA^®^ KS 400 ic control, IKA^®^-Werke GmbH and Co. KG, Staufen im Breisgau, Germany) at 70 rpm at room temperature for 48 h. After loading, BNC fleeces were transferred into 100 mL ultrapure water and shaken at 70 rpm and room temperature for a further 24 h to enable PLGA precipitation. The water was replaced after 4 and 8 h to ensure sufficient solvent removal.

The water-dependent phase transition from PLGA dissolved in the different solvents was determined by gradually adding ultrapure water to the respective loading solutions. The optical density at 600 nm was determined using a GENESYS 10 UV-Vis spectrophotometer (Thermo Fisher Scientific Inc., Waltham, MA, USA) after each dilution step. The water concentration, at which a significant change in optical density compared to the water-free polymer solution could be detected, was considered the critical water concentration for phase inversion.

### 2.3. Scanning Electron Microscopy (SEM)

PLGA-loaded fleeces were freeze-dried at −30 °C and 0.011 bar for 72 h using an Epsilon 2-4 LSC (Martin Christ GmbH, Osterode am Harz, Germany). After drying, cross-sections of the fleeces were cut and mounted on a SEM sample holder. Samples were sputtered with 4 nm platinum (CCU-010 HV Compact Coating System, safematic GmbH, Bad Ragaz, Switzerland). Microscopy was carried out using a Sigma-VP-Scanning Electron Microscope (Carl Zeiss AG, Oberkochen, Germany) operating at 4 kV using the in-lens detector. Particle size distribution was determined by calculating the equivalent spherical diameter of manually selected particles using ImageJ 1.52a (Wayne Rasband, National Institutes of Health, Bethesda, MD, USA).

### 2.4. Transparency

Transparency measurements were performed as described before [41], using the Tecan 20M multiplate spectrophotometer (Tecan Group AG, Maennedorf, Switzerland). PLGA-loaded and native BNC fleeces with a similar height were placed in 24-well plates (Greiner bio-one GmbH, Frickenhausen, Germany) and measured at a wavelength of 600 nm. The mean of the transmission of five equally distributed measurement points was calculated for each BNC fleece using the SparkControl™ 2.2. software (Tecan Group). Experiments were performed in triplicates and repeated once.

### 2.5. Fourier Transform Infrared (FT-IR) Spectroscopy

To verify the incorporation of PLGA, freeze-dried PLGA-loaded BNC was compressed and analyzed using a Nicolet iS5 FT-IR spectrometer equipped with an iD5 ATR unit (Thermo Fisher). For each sample, 16 spectra were recorded between 400 and 4000 cm^−1^ with a resolution of 0.482 cm^−1^. The spectra of PLGA-loaded fleeces were compared to the spectra of native BNC, PLGA, Sudan III, and the used solvent.

### 2.6. Differential Scanning Calorimetry (DSC)

Differential scanning calorimetry was used to characterize the influence of the precipitation and the used solvents on the thermal behavior of the PLGA phase. Measurements were performed by placing air-dried (48 h, 25 °C) BNC samples into aluminum pans and applying for a heating program from −10 °C to 250 °C with a heating rate of 10 °C min^−1^ on a Discovery DSC 250 (TA Instruments Inc., New Castle, DE, USA). The heating program was run in three cycles for each sample and TRIOS v4.3.1.39215 software (TA Instruments) was used for analysis. The heat flow curves of PLGA-loaded fleeces were compared to spectra of native BNC, bulk PLGA, and the applied solvent.

### 2.7. Compression Stability

Compression stability of native BNC and PLGA-loaded fleeces was characterized regarding weight and height before and after compression with a 400 g weight for 10 min according to Alkhatib et al. [8]. The compressive strain (*ε*) was calculated based on Equation (1):

*ε* = Δ*h*/*h*_0_ ∗ 100% (1)

where Δ*h* represents the reduction of height during compression and *h*_0_ the height before the compression of the fleeces. The mass reduction was expressed relatively according to Equation (2):

*m_loss_* = Δ*m*/*m*_0_ ∗ 100% (2)

where Δ*m* represents the reduction of mass during compression and *m*_0_ the mass before the compression of the fleeces. Experiments were performed in triplicates and repeated once.

### 2.8. Water Binding Characteristics

To evaluate the effect of the solvent treatment and PLGA precipitation on water-binding properties, water absorption value (WAV) and water retention value (WRV) were characterized according to Karl et al. [41]. For determining the WAV, PLGA loaded and native BNC were cut into quarters and re-swollen in the respective loading solutions for 2 h. The obtained pieces were dried at room temperature until they reached constant weight. The mass of the quarters was determined before and after drying and WAV was calculated according to Equation (3):

WAV = *W_i_*/*W_d_* ∗ 100% (3)

*W_i_* represents the initial weight before drying and *W_d_* is the weight after drying. The WRV was determined by cutting two cuboids with similar volumes out of a BNC fleece. The cuboids were re-swollen analogously to the WAV samples and subsequently centrifuged in a MiniSpin^®^ centrifuge (Eppendorf, Hamburg, Germany) for 15 min at 1073× *g*. The samples were dried at room temperature until they reached the constant weight and their mass was determined before and after drying. The WRV was calculated according to Equation (4):

WRV = (*W_i_* − *W_d_)*/*W_d_* ∗ 100% (4)

where *W_i_* represents the weight of the samples in the wet state after centrifugation and *W_d_* represents the weight of the dried samples. Experiments were performed in triplicates and repeated once.

### 2.9. Fluid Release

Native and PLGA-loaded BNC were investigated for their fluid-releasing capabilities using a method previously reported by Zahel et al. [42]. Briefly, an aqueous solution containing 142 mmol L^−1^ sodium chloride as well as 2.5 mmol L^−1^ calcium chloride (both Carl Roth) was prepared. Subsequently, 35% (*w*/*w*) gelatin (Bloom 180, Carl Roth) was suspended in this solution and incubated at 60 °C until fully dissolved. Afterward, Petri dishes with a diameter of 100 mm were filled with 37 g of the gelatin solution each, tightly sealed, and incubated at 25 °C for 3 h to allow the gelation of gelatin. Subsequently, the Petri dishes were weighed, the BNC samples were placed on the gelatin surface afterward and the dishes were tightly sealed. After 48 h of incubation, the BNC samples were removed, and the dishes were weighed again. The fluid release (*FR*) was calculated according to Equation (5):

*FR* [g/100 cm^2^] = (*m_after_* − *m_before_*) ∗ 100/*A* (5)

where *m_after_* represents the weight of the Petri dishes after incubation, *m_before_* the weight of the Petri dishes before incubation, and *A* the contact area between BNC fleece and gelatin gel. Experiments were performed in triplicates and repeated once.

### 2.10. High-Performance Liquid Chromatography (HPLC)

CBD (kind gift of Kannaswiss AG, Kölliken, Switzerland) was quantified using a 1260 Infinity II HPLC equipped with the 1260 quaternary pump, 1260 vial sampler as well as the 1260 variable wavelength detector (Agilent Technologies, Inc., Santa Clara, CA, United States). The software OpenLAB CDS Rev. C.01.07 (Agilent) was used for method control and analysis. The mobile phase was composed of mixtures of acetonitrile, 0.1% phosphoric acid, and methanol in different volumetric ratios (mobile phase A: 55:40:5; mobile phase B: 85:10:5) and a linear gradient according to Table S1 was used while maintaining a flow of 1.5 mL min^−1^. An XTerra RP18 column with 5 µm pore size, 4.6 mm diameter, and 250 mm length (Waters Corporation, Milford, MA, USA) was used and tempered to 60 °C. An injection volume of 20 µL was conducted for each run and detection by absorbance was performed at 225 nm. A linear calibration between 1.0 and 100.0 µg mL^−1^ CBD was performed in triplicates. The limit of detection and limit of quantification were calculated based on the standard deviation of the response and the slope of the calibration curve according to the International Council for Harmonisation of Technical Requirements for Pharmaceuticals for Human Use (ICH) Quality Guideline Q2 (R1) [43]. The HPLC quantification of AKBA was performed according to Karl et al. [41], a detailed description can be found in the Supplementary Information (S.2.1).

### 2.11. Drug Loading and In Vitro Release

Formulation approaches that were considered suitable for the evaluation as drug delivery systems were investigated regarding the loading and release of CBD or AKBA. A total of 1.0 mg mL^−1^ CBD was added to the polymer solution instead of Sudan III. Loading and precipitation were performed as described in Section 2.2. The amount of loaded CBD was determined by measuring the concentration of the loading solution before and after the loading process via HPLC as described before [44]. Additionally, the water used for the precipitation was probed after the finished process and analyzed for the occurrence of CBD by HPLC. Vertical Franz diffusion cells (Gauer Glas, Püttlingen, Germany) were used to investigate the release behavior of the loaded fleeces, as described before [45]. Loaded BNC fleeces were placed in the donor compartment of the cells and fixed with a thin defined aluminum foil cross, resulting in a direct contact area of 1.48 cm^2^ between the BNC fleece and release medium. A polyolefin wax membrane (PARAFILM^®^ M, Carl Roth) was used to cover the donor compartment. A total of 12 mL of phosphate buffered saline supplemented with 6% Brij^®^ O20 as well as 0.1 mg mL^−1^ tocopherol were used as release medium under magnetic stirring at 110 rpm and tempering to 32 °C. A total of 400 µL aliquots of the release medium were taken after defined time points (0, 0.25, 0.5, 1, 2, 4, 8, 12, 24, 48, 72, 96, 120, 144, 168, 192, 216, 240, 312 and 336 h) and replaced with fresh medium. Tocopherol was further added to stabilize the released CBD in the release medium, which was necessary over the long test period, as it is susceptible to oxidative degradation in aqueous media [46]. Samples were analyzed by HPLC for CBD quantification. The solubility of CBD in the release medium was determined to ensure sink conditions. The concentration of CBD in the supernatant of a saturated solution in the release medium was determined by HPLC after centrifugation at 13,500 rpm for 5 min. Experiments were performed in triplicates and repeated once. As a comparison, BNC loaded with 1 mg mL^−1^ CBD dissolved in propylene glycol by the submerse absorption technique [44] was released in the same setup. The loading and release of AKBA were carried out in a similar manner, a detailed description can be found in the supplementary information (S.2.2). The results were mathematically modeled by applying different mathematical models for characterizing the release properties, including Ritger and Peppas’ semi-empirical power-law [47] (Equation (6)), first-order kinetics (Equation (7)), zero-order kinetics (Equation (8)), or the Higuchi square root equation [48] (Equation (9)).

*M_t_*/*M_∞_* = *k* ∗ *t^n^* (6)

Ln*M_t_ =* Ln*M_*0*_ + k* ∗ *t* (7)

*M_t_ = M_*0*_ + k* ∗ *t* (8)

*M_t_ = k* ∗ *t*^1/2^ (9)

where *M_t_* represents the cumulative amount of released drug at time *t*, *M*_0_ *is* the initial amount of released drug, and *M_∞_* the released amount at the equilibrium state. Results were fitted between 5 and 60% of the maximum released amount of drug for each equation. For the Ritger-Peppas equation, the diffusional exponent *n* was determined by plotting the logarithmic amount of released drug against the logarithmic time and determining the slope of the resulting linear relationship.

### 2.12. Data and Statistical Analysis

The testing for statistical significance was performed by t-test and ANOVA one-way analysis of variance combined with the Tukey post hoc test using OriginPro 2019 (Originlab, Wellesley Hills, MA, USA). Differences were considered statistically significant with *p*-values < 0.05.

## 3. Results and Discussion

### 3.1. Development of the In Situ Inversion for Selection of Suitable Solvents

**Figure 1:**
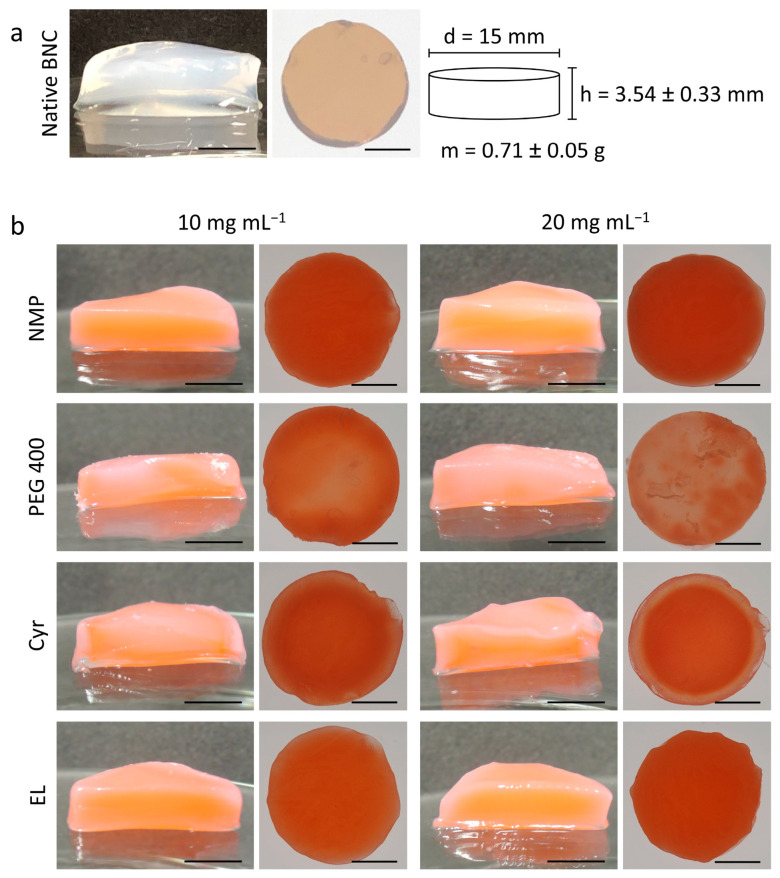
(**a**) Macroscopic cross-section and top view with the backlight of native BNC after synthesis and purification (scale bars = 5 mm), schematic depiction of diameter, height, and weight (mean ± SD, *n* = 50) of the used fleeces. (**b**) Macroscopic cross-section and top view with the backlight of BNC fleeces after in situ precipitation of PLGA using different organic solvents containing the lipophilic dye Sudan III (scale bars = 5 mm).

The biotechnological nanocellulose production performed by the *K. xylinus* culture led to the formation of homogenous BNC layers at the interface between air and culture medium. After purification and punching, white, homogenously turbid, and cylindrical BNC fleeces with a diameter of 15 mm, a mass of 0.71 ± 0.05 g and a height of 3.54 ± 0.33 mm could be obtained (Figure 1a). A volume of 0.62 ± 0.05 cm^3^ and a surface area of 5.20 ± 0.12 cm^2^ could be calculated based on the dimensions of the fleeces. A profound analysis of the physiochemical material characteristics of the BNC produced this way has already been described in a previous publication [39]. The material produced by this method is a hydrated three-dimensional polymeric network consisting of fibrils with diameters between 20 and 100 nm and pore diameters between 2 and 4 µm [39,49]. The material can be produced from sustainable raw materials and is already crosslinked, which is an advantage over synthetic hydrogels that require physical or chemical procedures under harsh conditions or reactive chemicals that can potentially impair their biocompatibility [50]. The small pore diameters make absorption of preformed polymer microparticles impossible, necessitating the process of in situ formation [10].

In contrast to previous studies [51,52] describing the combination of nanocellulose with polyesters, the approach presented in this study was aiming to establish microparticles embedded in BNC for the delivery of lipophilic drugs rather than modifying the mechanical characteristics of the material. Besides its common use in the formulation of nano- and microparticles, the ester-terminated Resomer RG502 was chosen as a standard polymer for the release of lipophilic drugs [53]. The comparatively low molar mass of the polymer was estimated to have a low impact on the viscosity of the loading solutions, enabling a sufficient loading of BNC with the polymer. PLGA particle preparation by phase inversion requires polymer dissolution in a suitable solvent miscible with a non-solvent (usually water) to allow a rapid change in polymer solubility for particle precipitation.

As a conventional standard solvent for the dissolution of PLGA NMP was selected that has been described for PLGA nanoparticle formulation via nanoprecipitation [54] and as a solvent for injectable in situ forming PLGA implants of already approved formulations [55]. NMP is controversially discussed due to its irritating effects and reproductive toxicity [56,57] as well as potential environmental hazards [58]. Alternatively, three alternative solvents, namely PEG 400, Cyr, and EL were investigated for the in situ formation of particles within BNC. Besides its use in injectable, oral, or topical formulations [59] the low molecular weight polymer PEG 400 has already been proposed as an alternative solvent for PLGA nanoprecipitation [60,61] and in situ forming PLGA implants [62]. The aprotic solvent Cyr is biodegradable, non-mutagenic, and can be obtained in a simple two-step synthesis from the renewable resource cellulose with low environmental impact [63]. The applicability of Cyr for PLGA nanoparticle formulation has previously been investigated by Grune et al. [64]. EL can be synthesized from renewable resources, is biodegradable due to its hydrolyzable ester functionality [33], and was described for the manufacturing of PLGA foams [65].

Table 1 summarizes the relevant physicochemical characteristics of the selected solvents. Whereas NMP, Cyr, and EL exhibit a low molar mass and viscosity, PEG 400 shows distinctly higher values for both parameters. All solvents were able to dissolve PLGA in concentrations of 10 and 20 mg mL^−1^ but differed in their respective critical water concentration for phase inversion. While PLGA solutions in NMP or Cyr exhibited a relatively high tolerance for water with values between 14 and 17% (*v*/*v*), PEG 400 and EL exposed noticeably lower critical concentrations of 6 to 7% (*v*/*v*) and 1 to 2% (*v*/*v*), respectively.

Since the proposed loading method aimed to incorporate highly lipophilic small molecules into BNC, the lipophilic red dye Sudan III (molecular weight = 352 g mol^−1^, log P = 5.7 [70]) was used as a model compound to evaluate drug distribution. The labeling of the samples refers to the used solvent and the PLGA concentration, e.g., NMP 10. The loading of the polymer solutions was performed using a post-synthesis loading technique based on the immersion of BNC fleeces in PLGA solutions as shown before for hydrophilic polymers [71] as well as water-insoluble polymers such as poly(lactic acid), poly(caprolactone), cellulose acetate, or poly(methyl methacrylate) using supercritical carbon dioxide as solvent [51]. Since the distribution of the polymer solution into the BNC network is mainly driven by diffusional processes, the different viscosities of the solvents have to be considered and a 48-h incubation time was chosen to ensure complete loading for all compositions. After loading, all fleeces appeared translucent. For PEG 400, large polymer aggregates outside of the fleece could be found, presumably since the critical water concentration for phase inversion was already exceeded due to the additional water bound in the BNC network. This was also observed for EL, but no large aggregates could be found.

In typical particle formulations, the precipitation of the polymer is induced by the removal of the solvent by evaporation or diffusion under stirring [17]. In the BNC matrix, the diffusive solvent exchange was induced by the transfer of the fleeces into ultrapure water und mild shaking. For all solvents, the phase inversion could be observed by the clouding of the loaded fleeces shortly after the transfer. To ensure complete polymer precipitation and solvent removal, fleeces were immersed in ultrapure water for 24 h with two complete water exchanges after 4 and 8 h.

The macroscopic inspection of the loaded fleeces gave some information about the distribution of the model drug Sudan III (Figure 1b) and differed depending on the selected solvent, but was independent of the PLGA concentration. The use of NMP and EL resulted in fleeces with intensive and homogenous red staining, which can be explained by a rapid solvent efflux due to their low viscosity and small molar size (Table 1). Even though NMP and EL differed greatly in their critical water concentration it seemed to have no major influence on the macroscopic appearance of the respective fleeces. In contrast, the use of PEG 400 led to a weaker staining, inhomogeneous dye distribution, and the occurrence of large white PLGA precipitates on the surface of the BNC fleeces, especially for PEG 400 with 20 mg mL^−1^ PLGA, which correlates with the high molar mass and viscosity of the solvent resulting in a low diffusivity. Although Cyr homogenously stained the fleeces, the formation of a brighter ring could be observed. This could be due to the formation of a temporary two-phase system already described by Grune et al. [64] for nanoprecipitation from Cyr solutions. As a result, Cyr very slowly mixed with the aqueous phase and the majority of polymer and dye precipitated at the phase boundary, leading to the formation of the visible ring within the fleeces.

**Figure 2:**
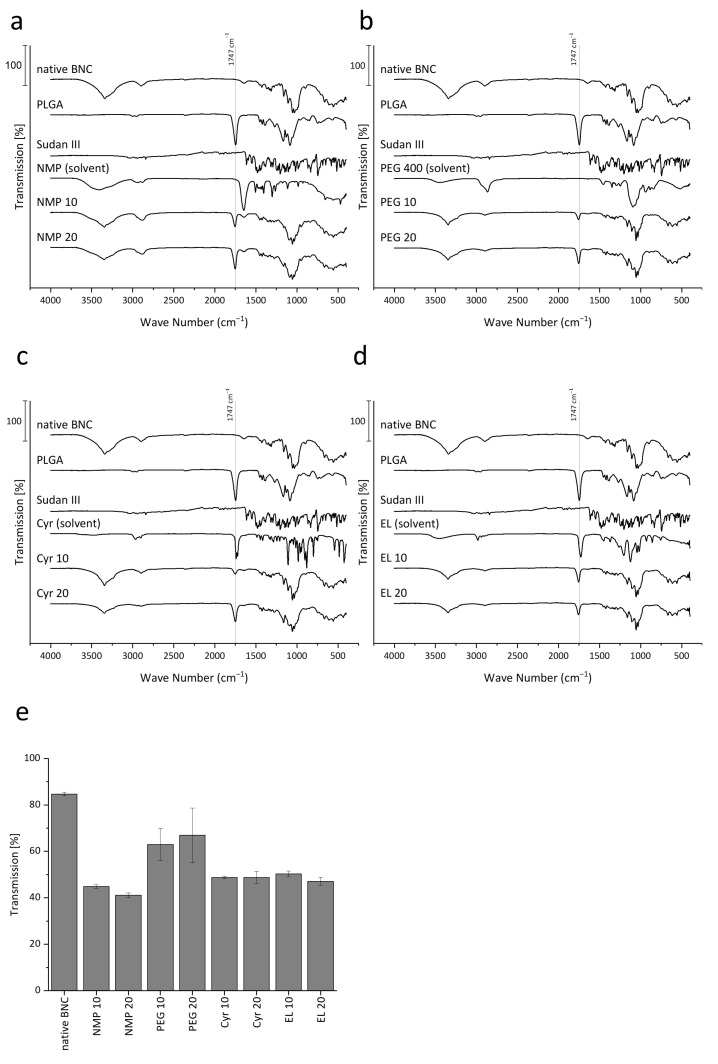
(**a**–**d**) ATR-FT-IR spectra of PLGA loaded fleeces in comparison to native BNC, Sudan III, pure PLGA, and the used solvents. Spectra are combined for the respective formulation approaches based on NMP (**a**), PEG 400 (**b**), Cyr (**c**), and EL (**d**). The marked bands at 1747 cm^−1^ are related to the stretching of the carbonyl functionality of PLGA. (**e**) Concentration and solvent-dependent light transmission through native BNC in comparison to PLGA loaded fleeces expressed as percentage transmission at 600 nm from five equally distributed points per fleece using UV/vis spectrophotometry (mean ± SD of six different fleeces from two independent experiments).

The presence of PLGA in the BNC matrix was confirmed by comparison of the FT-IR spectra of the PLGA-loaded fleeces with the single components (Figure 2a–d). The spectrum of native BNC showed characteristic signals with a broad characteristic band between 3000 and 3750 cm^−1^ related to the O-H-stretching of residual bound water and hydroxyl groups of cellulose, a band at 2900 cm^−1^ related to C-H-stretching and a band at 1645 cm^−1^ caused by O-H-bending of absorbed water. While these bands were also present in the PLGA-loaded fleeces, the occurrence of a band at 1747 cm^−1^ related to the stretching of the carbonyl functionality of PLGA confirmed the presence of the polymer regardless of the used solvent. Although NMP, Cyr, and EL also include a carbonyl functionality, their respective bands are shifted in comparison to PLGA, making it distinguishable from the PLGA-related signal at 1747 cm^−1^. No other additional bands could be detected, indicating that possible solvent residues are present only in minor concentrations, which implied suitable purification conditions. Although later for biological applications it would be necessary to quantify the residual solvent content, with respect to the dilution factor (1:500) during purification and the low toxicity of the solvents with LD_50_ values described above, acceptable biocompatibility could be suggested. This is also confirmed by earlier studies where around 2% residual solvents were detected for the preparation of nanoparticles using the same alternative solvents [61,64].

Additionally, the presence of PLGA particles in the BNC could also be indirectly confirmed by spectrophotometric investigation of BNC transparency at 600 nm (Figure 2e). An interference of Sudan III (absorbance maximum 506 nm) could be excluded in preliminary experiments (Figure S1). While native BNC revealed a transmission of 85%, on the other hand, it was reduced to comparable values around 40 to 50% for NMP, EL, and Cyr independent of the PLGA concentration, which indicates the formation of a disperse polymer phase in the BNC network as observed earlier also for nanoemulsions [41]. The inhomogeneous loading observed with PEG 400 as solvent was also represented by the higher transmission with high variability. The best loading results could be obtained by the use of NMP and EL demonstrating (i) fast phase transition, (ii) homogenous loading, and (iii) the ability to incorporate high amounts of polymer.

### 3.2. Morphology and Thermal Characteristics of the Polymer Containing BNC

**Figure 3:**
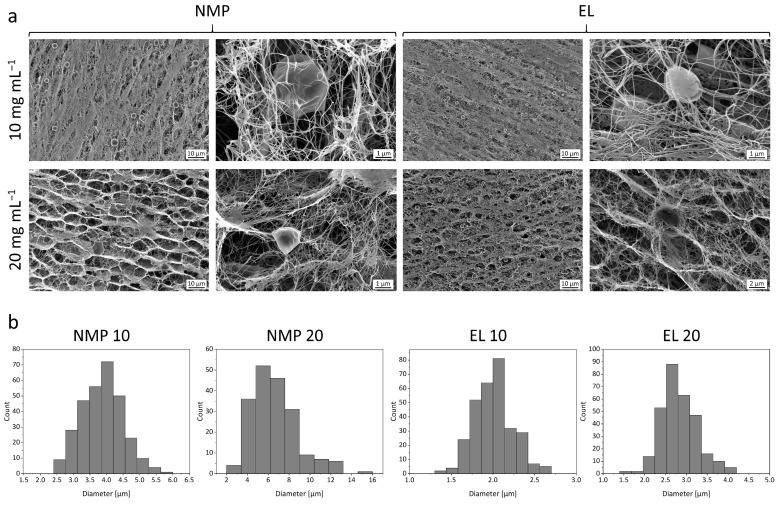
(**a**) Morphology of the PLGA phase within BNC depicted by scanning electron microscopy (1000 to 10,000× magnification) of the cross-section of the fleeces after precipitation of 10 and 20 mg mL^−1^ PLGA from the solvents NMP and EL and subsequent freeze-drying. (**b**) Distribution of the spherical equivalent diameter of PLGA particles incorporated into BNC determined by ImageJ (*n* = 300 for NMP 10, EL 10, and EL 20, *n* = 192 for NMP 20).

The morphology of BNC with incorporated PLGA and Sudan III was investigated via scanning electron microscopy. The electron-microscopic view revealed major differences in the structure of the polymer phase for the different solvents (Figure 3a). For the case of NMP 10, the precipitation of spherical particles in the micrometer range could be observed, NMP 20 contained microparticles as well, but with a highly irregular shape. The use of EL led to the formation of a larger number of homogenously distributed, spherical particles at both concentrations, with a few observable aggregates for EL 20. Higher magnifications showed that the particles were often associated with cellulose fibrils. The surface of the PLGA particles derived from NMP and EL solutions was smooth and without observable pores. In accordance with the macroscopic appearance, only very few singular particles could be found inside the BNC network for PEG 10 and PEG 20, which highlights the hypothesis that the majority of the polymer precipitates on the outside of the BNC fleece under the chosen conditions. Whereas some single particles could be found for fleeces treated with Cyr 10, for Cyr 20 a few large aggregates interconnecting BNC fibrils could be observed (Figure S2). Apparently, the conditional water miscibility of Cyr did not enable efficient spontaneous precipitation of spherical particles. Although this hurdle can be overcome by high-energy ultrasonification, as demonstrated for the precipitation of PLGA nanoparticles from Cyr solutions [64], this approach is not applicable to BNC matrices. Consequently, Cyr was not further considered for the formation of polymer microparticles within BNC and the formation of spherical polymer particles within BNC by the in situ method should be performed using fully water-miscible solvents with low viscosity and low molecular weight, such as NMP and EL. Additionally, all electron micrographs were checked for drug residual, but in none of the investigated fleeces any crystalline precipitations were found.

The histograms in Figure 3b allow the comparison of the size distribution of the microparticles obtained by phase inversion, determined as equivalent spherical diameter. Mean particle sizes of 3.87 ± 0.62 µm for NMP 10, 6.63 ± 2.23 µm for NMP 20, 2.01 ± 0.24 µm for EL 10 and 2.82 ± 0.43 µm for EL 20 revealed an increase of size with higher polymer concentrations. An increased tendency for particle nucleation and aggregation up to the uncontrolled formation of a coherent polymer network was suggested to be responsible for this effect as observed earlier by Pircher et al. [51], who investigated the precipitation of different polymers within BNC using supercritical carbon dioxide as a solvent. Particles precipitated from EL solutions were considerably smaller compared to NMP which might be related to the different critical water concentrations for phase inversion. The higher tolerance of NMP-based PLGA solutions for water promotes extended particle growth due to a longer time frame for particle nucleation and aggregation resulting in larger particle diameters. The influence of the critical water concentration for precipitation on the final size distribution was earlier reported for the solvents *N*,*N*-dimethylacetamide, *N*,*N*-dimethylformamide, and tetrahydrofuran for the nanoprecipitation of polystyrene via dialysis. Higher mean particle sizes were obtained for solvents with higher water tolerance [72].

Although the post-synthesis loading of BNC with pre-formulated drug-containing particles by adsorption has been reported for poly(ethylene oxide)-b-poly(caprolactone) [4], nanochitosan dots [5], zein [6] and hyperbranched polycationic polysaccharides [7], for the realization small particle sizes of 200 nm or less are necessary to achieve sufficient penetration into the dense BNC network. In contrast, the in situ phase inversion enables the combination with larger polymer microparticles more suitable for realizing the controlled release of therapeutic compounds [9], which broadens the application spectrum of BNC. To accomplish a homogeneous distribution of the particles in the BNC network, the one hand, fleeces with highly standardized dimensions and fibril densities have been used [39]. On the other hand, shaking conditions have been selected that are known from previous studies [40,44] to efficiently and completely distribute even larger materials in fleeces of the selected dimensions [41].

**Figure 4:**
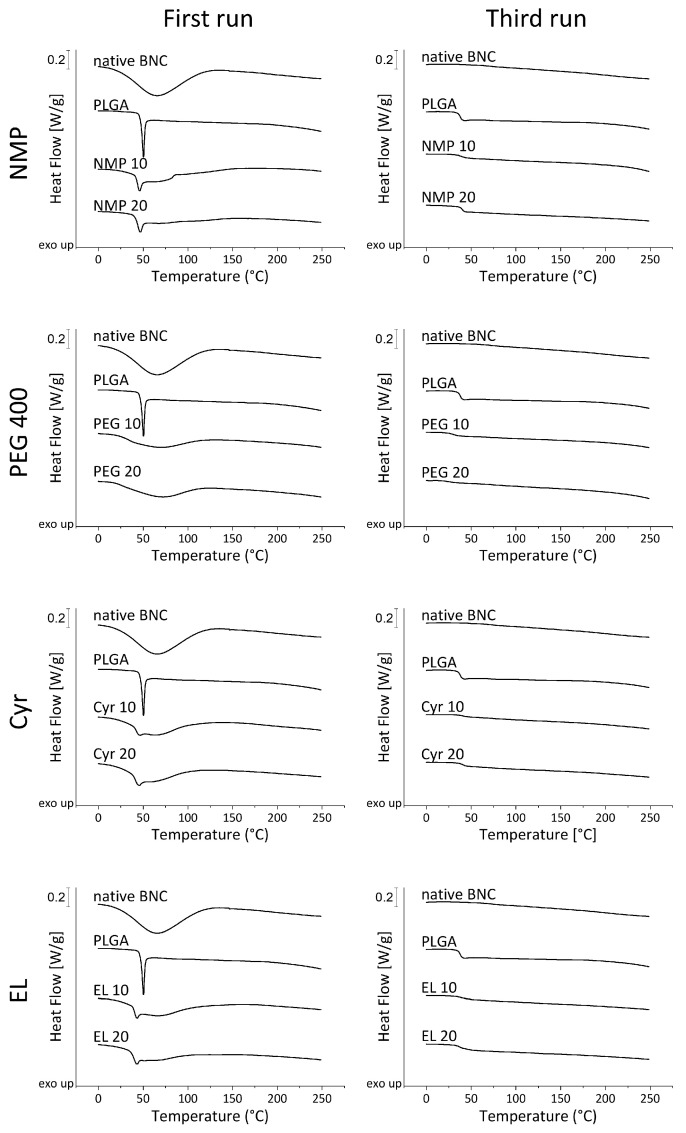
Differential scanning calorimetry of PLGA loaded fleeces compared to native BNC and bulk PLGA. Three heating-cooling cycles between 0 and 250 °C at 10 °C min^−1^ were applied; heat flow curves of the first and third heating cycles are depicted.

The determination of thermal properties, especially the glass transition temperature, reveals information on polymer crystallinity. DSC measurements investigated the thermal properties and crystallinity of BNC and PLGA alone and in combination with heat flow curves to identify differences resulting from the use of different solvents (Figure 4). In the first run, native BNC demonstrated a wide endothermic peak between approximately 5 and 120 °C related to the evaporation of residual water. The third run revealed the high thermal stability of BNC without noticeable signals up to 250 °C, which is in agreement with prior reports of decomposition temperatures about 290 °C [73] to 330 °C [74]. PLGA exhibited a sharp endothermic peak at 50 °C in the first run related to the melting of the polymer, comparable to earlier results [64]. This indicates the presence of crystalline regions within the PLGA microparticles. However, due to an overlap of the signal with that of the evaporating water, it was not possible to analyze phase transition enthalpy or degree of crystallinity. The glass transition temperature could be detected in the third run with a value of 38 °C. No shift of the glass transition temperature could be observed when using NMP and EL, which indicates that PLGA did not hydrolyze during the formulation procedure as a decreasing molecular weight would lead to lower glass transition temperatures [75]. While fleeces treated with Cyr showed similar characteristics, the particles obtained by precipitation from PEG 400 showed no melting point and a glass transition temperature shifted to lower temperatures between 25 and 30 °C. The signals are weak compared to the other solvents presumably because of the low amount of incorporated polymer, nevertheless, the results are plausible since PEG 400 is known to act as a plasticizer for PLGA [76].

Based on the results of the physicochemical characterization, requirements for solvents used for the in situ formation of polymeric particles within BNC could be concluded. The used solvent should exhibit unrestricted miscibility with water to ensure a fast phase inversion and the easy removal of solvent residues. Additionally, a low molecular weight and a low viscosity are preferable to accomplish efficient loading of the polymer into BNC and to enable a fast phase inversion of the dissolved polymer which translates to narrow particle size distributions and minimizes drug losses during the precipitation step. The critical water concentration for phase inversion should be evaluated for choosing an appropriate solvent since it impacts the final particle size within the fleeces. Potential plasticizing effects of the solvent should be considered as they can impact drug release. Finally, sustainability and biocompatibility should be considered to minimize the environmental impact and to ensure safe manufacturing and application of the drug delivery system.

### 3.3. Interaction with Water and Mechanical Characteristics of Microparticle Containing BNC

Based on the results from Section 3.1 and Section 3.2, only NMP and EL were selected as solvents for further investigations. When aiming for application as wound dressing, the formulation technique should have no or only a minimal impact on water binding and mechanical characteristics of BNC.

**Figure 5:**
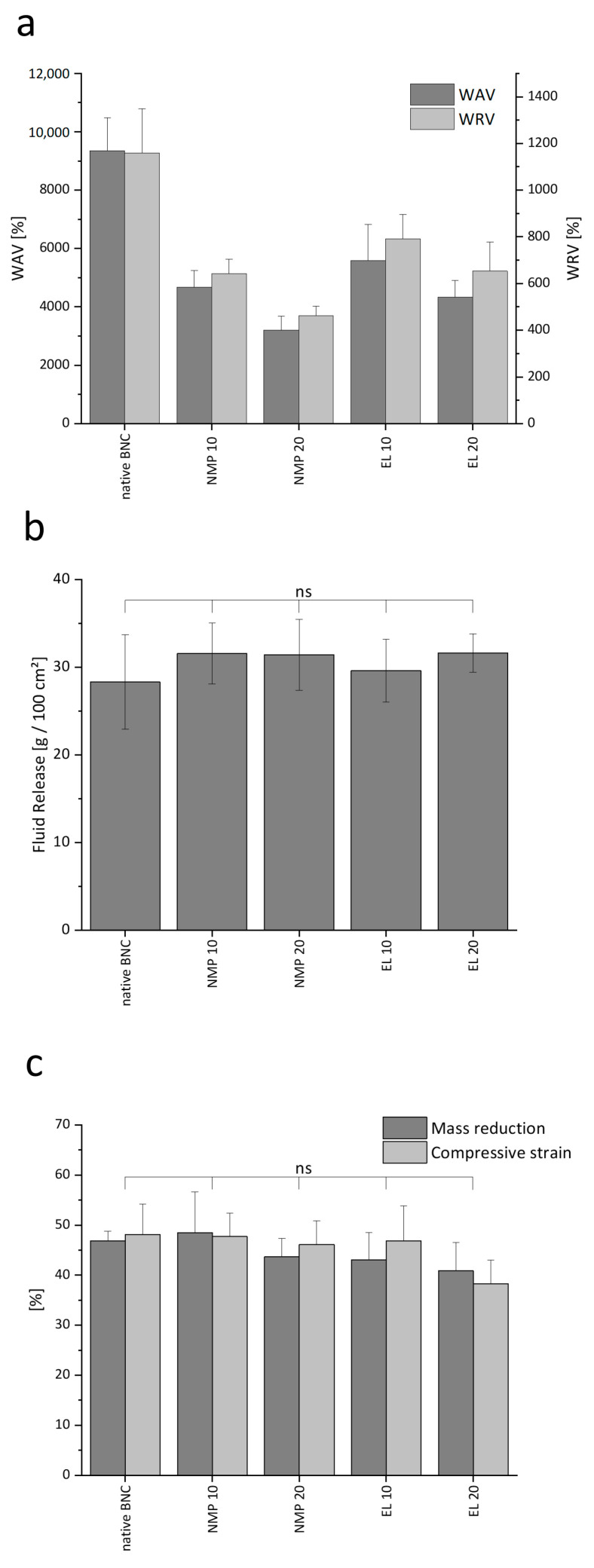
Comparison of the physicochemical characteristics of native BNC and PLGA loaded fleeces with (**a**) WAV and WRV, (**b**) fluid release, and (**c**) compression stability (mean ± SD of six different fleeces from two independent experiments, ns marks non-significant differences compared to native BNC with *p* > 0.05).

The outstanding water-binding capacity is a key advantage of BNC as demonstrated by the promotion of the healing process, especially in chronic wounds due to the moisturizing environment [77]. The WAV represents the total amount of water bound by the cellulose network expressed relative to the solid content. For native BNC a WAV of 9356 ± 1129% was obtained, which is in line with prior reports for the material produced in scale-up [39] (Figure 5a). In comparison, the loading with lipophilic PLGA reduced the WAV of the material in a polymer concentration-dependent way (3200–5600%), depending on the type of solvent. The WRV was used to express the water binding of the material during compression, simulated by centrifugal force (Figure 5a). Whereas for native BNC a value of 1160 ± 189% was obtained, comparable to earlier results [8,41], the PLGA incorporation resulted in a reduction of the WRV with higher polymer concentrations. The use of NMP led to higher reductions in WAV and WRV than EL. This indicates higher polymer incorporation rates for NMP since the amount of water bound by the dry material decreases with higher polymer content. The observed reduction of WAV and WRV by the incorporation of lipophilic materials in the BNC matrix corresponds to earlier reports investigating formulation strategies for lipophilic drugs. Compared to the microparticle precipitation, the covalent binding of lipophilic residues such as hydrophobic amino acids or acetyl functionalities had less impact on the water binding characteristics [78], while the incorporation of nanoemulsions led to a higher reduction of WAV and WRV [41]. Therefore, the PLGA precipitation represents a middle ground between these techniques that enables the incorporation of lipophilic compounds with a comparatively low impact on water binding characteristics.

To simulate the fluid-releasing capabilities of the loaded BNC more closely to the physiological situation, the amount of liquid delivered from the BNC fleece to a gelatin gel [42] was determined (Figure 5b). Qualitatively, as shown in Figure S3, all gels were visibly swollen at the contact surface to all BNC samples after 48 h incubation. Quantitatively, native BNC and all particles containing BNC demonstrated average fluid release values between 28 and 32 g/100 cm^2^ without significant (*p* > 0.05) differences between the different formulation approaches. Conclusively, the fluid release was not impaired by the incorporation of PLGA particles into the BNC network or the reduction of WAV and WRV.

Another unique property of the BNC is its high-pressure stability [79] relevant for applications such as lymphatic drainage using pressure dressings or the mechanical stress during its lifetime through many processes, e.g., packaging. Compression could especially lead to a loss of the incorporated fluids by squeezing, limiting the hydrating capacity of the material. Therefore, compressive strain and weight loss of the PLGA-loaded fleeces were compared to native BNC (Figure 5c). The native material exhibited a weight loss of 47 ± 2% and a height reduction of 48 ± 6% due to a partial loss of water. No significant changes could be detected for any of the PLGA-loaded fleeces, meaning that the mechanical stability was not affected by solvent treatment and precipitation. The results are consistent with a report by Pircher et al. [51], who showed for poly(lactic acid) no relevant changes in the BNC compression stability up to a concentration of 80 mg mL^−1^. Considering the chemical similarity of PLGA to poly(lactic acid) and the low concentrations of 10 and 20 mg mL^−1^ in the present formulation approach, the absence of an effect on the pressure stability of BNC is plausible.

### 3.4. Potential of PLGA Loaded BNC as Drug Delivery System

To evaluate the applicability of the microparticulate formulation approach for drug delivery, drug loading and release studies from BNC were performed using the highly lipophilic drugs CBD and AKBA. CBD, a non-hallucinogenic compound from *Cannabis sativa* has excellent antimicrobial activity against gram-positive bacteria such as *Staphylococcus aureus* or *Clostridioides difficile* probably related to the disruption of bacterial membranes [37] while having a low tendency to induce resistances. Additionally, anti-inflammatory effects could be demonstrated [80]. AKBA, the pharmacologically active lead compound of *Boswellia serrata* has a strong anti-inflammatory effect caused by the allosteric modulation of 5-lipoxygenase (5-LOX), which shifts the product spectrum of the enzyme from pro-inflammatory to actively inflammation-resolving mediators [38]. The anti-infective and anti-inflammatory potential of CBD and AKBA both with a high, but different lipophilicity (CBD: log P = 6, AKBA: log P = 8) made them ideal candidates to investigate the feasibility of the developed formulation approach. For both drugs, the formulation within a long-acting hydrogel-based delivery system is desirable in applications such as active wound dressings or implants for tissue regeneration.

Due to their lipophilicity, the active ingredients were dissolved together with PLGA in each of the selected solvents and loaded into BNC to allow their encapsulation. The loading efficiency was evaluated based on the concentrations of the loading solutions before and after the loading process. Additionally, during polymer precipitation, the excess water was probed and analyzed to investigate the partitioning of the drugs into the water phase during that process. To compare the drug loading and release properties of systems based on the conventional solvent NMP and as well as the sustainable green solvent EL, experiments for CBD were performed with the formulations NMP 10, NMP 20, EL 10, and EL 20.

For CBD, comparable loading efficiencies of 9.65 ± 3.20%, 10.09 ± 1.96%, 9.86 ± 1.97%, and 10.47 ± 1.78% were determined for NMP 10, NMP 20, EL 10, and EL 20, respectively, without statistically significant differences (*p* > 0.05). These values correlated to the volume ratio of BNC and the loading solutions as observed before for many drug solutions and dispersions using the agitated submerse loading technique [40,44]. For further studies, to accomplish higher loading efficiencies lower volumes of more concentrated loading solutions, or partial removal of the initially bound water by mechanical pressure or forced evaporation could be taken into consideration. Furthermore, other loading techniques using dried BNC in a reswelling process would be interesting to investigate [81]. Obviously, the different types of solvents and varying polymer concentrations did not influence the final drug load under the chosen loading conditions. Additionally, no CBD could be found in the probed water from the precipitation process, indicating negligible drug losses during the precipitation step. Due to the comparability of the NMP and EL results for CBD, for AKBA, only the EL-based loading technique was applied. Comparatively low loading efficiencies of 6.13 ± 0.98% and 6.63 ± 1.19% were determined for EL 10 and EL 20, respectively. Apparently, due to the higher hydrophobicity of AKBA compared to CBD, a lower partition into the hydrophilic cellulose network took place for the boswellic acid during the loading step. During precipitation, again, no active ingredient was found in the aqueous supernatant.

**Figure 6:**
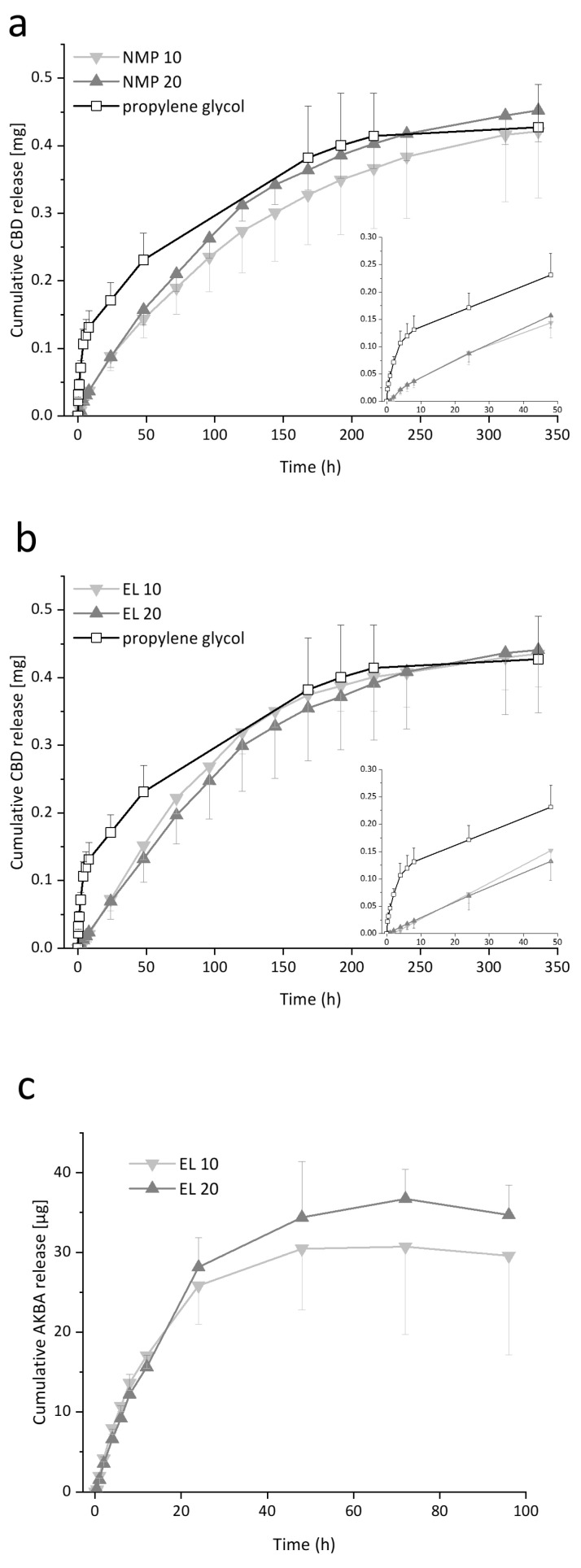
(**a**,**b**) Cumulative release of CBD [mg] from PLGA-loaded fleeces (NMP 10, NMP 20, EL 10, and EL 20) and propylene glycol-loaded fleeces over up to 336 h in vertical Franz diffusion cells. The inserted graph displays the same data over 48 h. (**c**) Cumulative release of AKBA [µg] from PLGA-loaded fleeces (EL 10 and EL 20) over up to 96 h at 32 °C in vertical Franz diffusion cells. Results are expressed as mean ± SD from six individual patches from two independent experiments.

Release experiments were conducted using vertical Franz diffusion cells with samples in direct contact with the release medium to relate to the unidirectional contact of BNC to the wound surface. In addition to the particle-containing fleeces, the release was also characterized for BNC-containing CBD dissolved in propylene glycol at the same concentration to directly compare the release kinetics of the different formulation approaches. The solubility of CBD in the release medium was determined as 4.77 ± 1.70 mg mL^−1^, ensuring sink conditions over the course of the experiment. Figure 6a,b depicts the cumulative release of CBD in relation to the determined drug load. All particles containing fleeces demonstrated a similar release behavior regardless of the applied solvent, polymer concentration, or particle size, with almost linear release rates up to 96 h, followed by a slower release until the equilibrium state was reached at 240 h. The released amounts of CBD varied between 0.42 and 0.45 mg, which corresponds to 45 to 48% of the incorporated drug. For comparison, fleeces containing CBD as propylene glycol solution demonstrated a fast biphasic release profile with an initial burst release within the first 8 h followed by a slower release up to 216 h, which is comparable to that of many drugs incorporated into BNC as a solution [44]. The difference between the particle and solution-based systems becomes particularly clear when the initial release behavior is considered. The encapsulation of the drug in microparticles within the BNC network minimized the initial burst release that is usually driven by the rapid dissolution of non-encapsulated or surface-adsorbed drugs [82]. A high encapsulation efficiency can be expected based on the high lipophilicity of the used drugs and the comparatively low drug-to-polymer ratios compared to previous publications reporting high encapsulation rates of CBD and AKBA in PLGA, e.g., in nanoparticles. Fraguras-Sánchez et al. [83] encapsulated CBD in PLGA nanoparticles with 78–90% encapsulation efficiency with a drug-to-polymer ratio of 1:5. For AKBA, Bairwa et al. [84] reported over 80% encapsulation efficiency for the encapsulation in PLGA nanoparticles also using a drug to polymer ratio of 1:5.

Up to now only a few reports of controlled release from BNC-based drug delivery systems were published, including the incorporation of gelling agents [8,85], the application of drying techniques [86], or the attachment of additional polymer layers [87]. To the best of our knowledge, the incorporation of PLGA in form of microparticles into BNC has not been investigated in this regard.

To evaluate the release process and gain insight into the underlying release mechanisms, the Ritger-Peppas model was applied, considering the BNC fleeces as a non-degradable, swellable polymeric cylinder (Table S2). The applicability of this model for modeling the release from hydrogels containing PLGA microparticles has been demonstrated by Garakani et al. [88] with the overall highest fit for the power law equation in comparison to other mathematical models. The release from CBD incorporated as a solution resulted in a diffusional exponent of 0.45 indicating an anomalous, non-Fickian transport mechanism and the BNC as release controlling matrix with an overlay of diffusion and swelling as described before in several reports for this type of incorporation strategy [40,44]. Higher diffusional exponents of 0.75–0.79 for NMP and 0.95–1.02 for EL were obtained with comparable values for both polymer concentrations indicating the influence of the PLGA microparticles. For EL the diffusional exponents >0.89 indicated a release profile with zero order kinetics. The difference between NMP and EL could be related to the different particle sizes and polydispersity. Larger particle sizes and the broader size distribution of microparticles derived from NMP apparently resulted in a slower release rate and slightly modified the kinetics. Even if this is noticeable when fitting the release curves with the power law, the slightly different release kinetics should be negligible in practical applications. Additionally, the release curves were fitted applying mathematical models for first-order release kinetics, zero-order release kinetics as well as the Higuchi square root equation (Table S3). The solution-based system showed the highest fit for the Higuchi square root equation, indicating a diffusion-driven drug release. For EL-based microparticulate systems, the best fit was obtained for zero-order kinetics, confirming the results obtained by applying Ritger and Peppas’ power law. NMP-based systems demonstrated similar fits with coefficients of determination >0.99 for both, zero-order kinetics and the Higuchi equation.

The release profile of the proposed systems is influenced by multiple simultaneous processes, including diffusion and swelling of the BNC, diffusion through the PLGA matrix, particle degradation through hydrolysis, swelling of the PLGA particles as well as diffusion of CBD through the aqueous pores. PLGA microspheres typically demonstrate an initial burst release by removal of surface-near drug deposits followed by a lag time with sustained release and a final fast release accelerated by polymer degradation [17]. In the present setting, the BNC matrix limited the direct contact of the PLGA microparticles with the release medium minimizing the initial burst release, which is a feasible explanation for the linear release kinetics from the proposed systems. This effect has already been reported for PLGA microparticles embedded in other hydrogels based on poly(vinyl alcohol) [89] or alginate [90] that also reported zero-order kinetics for the release of the incorporated drugs.

Particle degradation through hydrolysis also has to be taken into account over the investigated time frame of 10 days. Typically, autocatalysis for PLGA hydrolysis is less pronounced in particles in the lower micrometer range [75]. However, it cannot be completely excluded, especially considering the NMP-based systems with larger particle sizes. Galeska et al. [89] observed a reduction of particle degradation when incorporating PLGA microparticles within a poly(vinyl alcohol) gel matrix. The authors hypothesized a reduced partition of water from the hydrogel into the microspheres that reduced the hydrolytic ester bond cleavage. However, an evaluation of the particle degradation in the presented BNC-based systems and its effect on the release behavior is worth to be carried out in future studies.

Release experiments with the same setup were performed using the more lipophilic AKBA for the EL-based systems with a comparatively low amount of drug at a concentration of 0.3 mg mL^−1^ in the loading solution. Accordingly, the release process was completed more quickly in comparison to CBD within 48 h with similar release profiles for EL 10 and EL 20. Due to high variability differences in the released amount at the equilibrium state showed no statistical significance (*p* > 0.05 for 24, 48, 72, and 96 h). Nevertheless, as obtained for CBD, the release also followed zero-order kinetics according to Ritger and Peppas’ power law. Results for AKBA are presented in Figure 6c as well as Table S4.

Despite the potential use of the systems containing CBD or AKBA as active wound dressings other applications are possible as well. Since CBD is able to promote mesenchymal stem cell migration, the CBD-containing fleeces could be used for bone tissue repair, similar to the approach of Kamali et al. [91] who demonstrated the feasibility of a gelatin hydrogel containing PLGA microparticles with CBD for promoting bone regeneration. For AKBA, a similar application can be considered, since the boswellic acid is able to promote osteoblast differentiation by inhibition of tumor necrosis factors [92]. In contrast to other hydrogels, a crosslinking of BNC would not be necessary for this application, since the natural entanglement of cellulose fibrils results in the formation of mechanically stable pellicles during biosynthesis, which provides sufficient mechanical stability. This eliminates the need for using reactive chemicals that would otherwise have to be removed by complex purification procedures to ensure biocompatibility. Furthermore, since BNC, PLGA, and EL can all be synthesized from renewable resources [11,33,93], a manufacturing process fully relying on sustainable materials can be established for this drug delivery system, when using EL as solvent.

Even though the biocompatibility of the proposed systems has to be evaluated in further studies, some assumptions can be made based on their composition. For BNC fleeces the biocompatibility, even under long-term conditions, was demonstrated in various reports including cytotoxicity tests [94], hemocompatibility assays [95], and in vivo studies [96]. Similarly, biocompatibility data for PLGA were frequently published [97] and it is used as an excipient in various FDA and EMA-approved formulations [98]. Taking the available toxicological data of the solvents, discussed above, into consideration, the biocompatibility of the systems can be suggested.

## 4. Conclusions

In the present study, the extended and controllable release of lipophilic drugs from the highly hydrophilic BNC network was accomplished by PLGA microparticles formulated by in situ precipitation in the nanocellulose matrix. As a unique point, a phase inversion method was developed using different conventional and alternative solvents. Critical solvent characteristics such as water miscibility, viscosity, molecular weight, and the critical water concentration for precipitation were identified as parameters affecting the particle formation within BNC. In comparison to the conventional solvent NMP, only the green sustainable solvent EL enabled an equivalent formation of homogeneously distributed spherical PLGA microparticles within the cellulose network. The microparticle formation did not influence the favorable mechanical characteristics of BNC and had only a moderate impact on the water-binding characteristics of the material, which did not adversely influence the fluid release. Release experiments demonstrated the sustained release of the highly lipophilic drugs CBD and AKBA over up to 10 days with zero order kinetics when using EL as a solvent. Conclusively, the use of sustainable solvents enables the formulation of an entirely renewable composite material that could serve as a long-acting active wound dressing for the treatment of chronically inflamed wounds. Additionally, the ability to incorporate polymeric microparticles into BNC opens up numerous further possibilities for drug delivery and biomedical applications such as tissue engineering and regenerative medicine. It is anticipated that this proof-of-concept will be extended to other polymers and drugs, and biocompatibility and therapeutic efficacy will be investigated in the future.

## Figures and Tables

**Table 1:** Comparison of the physicochemical characteristics of the used solvents (* = experimental data).

Solvent		NMP	PEG 400	Cyr	EL
Structure		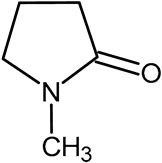	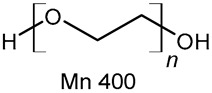	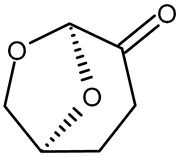	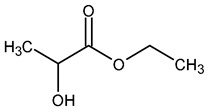
Molar mass [g/mol]		99	400	128	118
Viscosity at 25 °C [mPa s]		1.7 [66]	94.4 [67]	14.5 [68]	2.4 [69]
Critical water concentration for phase inversion [% *v*/*v*] *	10 mg mL^−1^ PLGA	15	7	17	2
	20 mg mL^−1^ PLGA	14	6	16	1

## Data Availability

The data presented in this study are available on request from the corresponding author. The data are not publicly available due to confidentiality concerns.

## Supplementary Materials

The following supporting information can be downloaded at: https://www.mdpi.com/article/10.3390/pharmaceutics15020559/s1, S.2.1 HPLC quantification of AKBA; S.2.2 Loading and in vitro release of AKBA; Figure S1: Absorption scan of the lipophilic dye Sudan III from 300–1000 nm showing the absorbance maximum at a wavelength of 506 nm. The scan was performed with a 0.1 mg mL^−1^ solution of Sudan III in 96% (*v*/*v*) ethanol using a Tecan 20 M multiplate spectrophotometer; Figure S2: Morphology of the PLGA phase within BNC depicted by scanning electron microscopy (1000 to 10,000× magnification) of the cross-section of the fleeces after precipitation of 10 and 20 mg mL^−1^ PLGA from the solvents PEG and Cyr and subsequent freeze-drying; Figure S3: Pictures of gelatin gels after 48 h of incubation in the fluid release experiments. The visibly swollen contact areas to the BNC fleeces are highlighted; Table S1: Gradient of mobile phase for CBD HPLC analysis; Table S2: Mathematical modeling of cumulative CBD release profiles applying Ritger and Peppas’ semi-empirical power-law; Table S3: Mathematical modelling of cumulative CBD release profiles applying first-order kinetics, zero-order kinetics and the Higuchi square root equation; Table S4: Mathematical modeling of cumulative AKBA release profiles applying Ritger and Peppas’ semi-empirical power-law.
