# Supplementary material for: In situ Formation of Polymer Microparticles in Bacterial Nanocellulose Using Alternative and Sustainable Solvents to Incorporate Lipophilic Drugs
Source: Pharmaceutics. 2023 Feb 7;15(2):559. doi: 10.3390/pharmaceutics15020559 (PMC9958971; doi:10.3390/pharmaceutics15020559)
Supplement: Supplementary file 1 [file pharmaceutics-15-00559-s001.zip › pharmaceutics-2160245-supplementary.pdf]

pharmaceutics

Supplementary Materials: In situ Formation of Polymer Microparticles in Bacterial Nanocellulose Using Alternative and Sustainable Solvents to Incorporate Lipophilic Drugs

Tom Bellmann, Jana Thamm, Uwe Beekmann, Dana Kralisch and Dagmar Fischer

S.2. Materials and Methods

S.2.1 HPLC quantification of AKBA

An isocratic method for AKBA quantification was adopted according to Karl et al. [1]. A 1260 Infinity II HPLC equipped with a Zorbax Eclipse 5 XDB-C18 (150 × 4.6 mm) column and a variable wavelength detector (all Agilent Technologies Inc., Santa Clara, CA, USA) were used for spectrophotometric quantification via absorption at 260 nm. The software OpenLAB CDS Rev. C.01.07 (Agilent) was used for method control and analysis. The mobile phase was composed of acetonitrile and 0.1% formic acid at fixed amounts of 90% and 10%, respectively. An isocratic flow of 1 mL min^{-1} was applied. An injection volume of 20 µL was conducted for each run. A linear calibration between 0.5 and 10.0 µg mL^{-1} AKBA (Rotichrom HPLC, Carl Roth GmbH + Co. KG) was performed, resulting in a correlation coefficient of 0.99990. The limit of detection and limit of quantification were calculated based on the standard deviation of the response and the slope of the calibration curve according to ICH Quality Guideline Q2 (R1) [2] with values of 0.16 and 0.48 µg mL^{-1}, respectively.

S.2.2 Loading and in vitro release of AKBA

0.3 mg mL^{-1} AKBA were added to the polymer solutions instead of Sudan III. Loading and precipitation were performed as described in section 2.2. The amount of loaded AKBA was determined by measuring the concentration of the loading solution before and after the loading process via HPLC, as described before [3]. Additionally, the water used for the precipitation was probed after the finished process and analyzed for the occurrence of AKBA by HPLC. Vertical diffusion cells (Franz cell, Gauer Glas, Püttlingen, Germany) were used to investigate the release behavior of the loaded fleeces, as described before [4]. Loaded BNC fleeces were placed in the donor compartment of the cells and 12 mL of phosphate buffered saline with 20% (V/V) ethanol were used as release medium under magnetic stirring at 110 rpm and tempering to 32 °C. 400 µL of the release medium was taken at defined time points (0, 0.25, 0.5, 1, 2, 4, 8, 12, 24, 48, 72 h) and replaced with fresh medium. Samples were analyzed by HPLC for AKBA quantification. Experiments were performed in triplicates and independently repeated once.

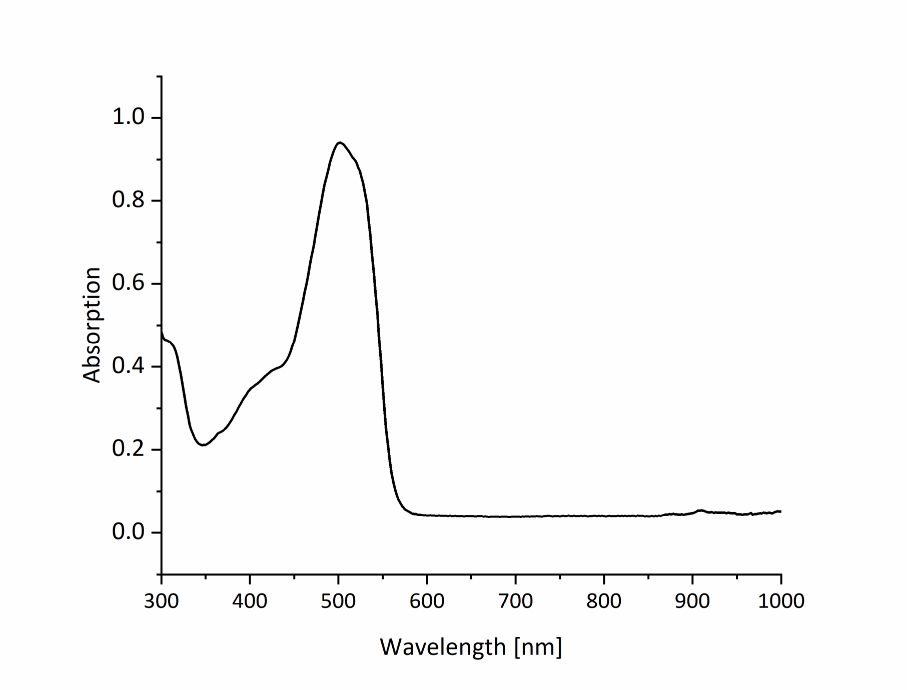

Figure S1: Absorption scan of the lipophilic dye Sudan III from 300 – 1000 nm showing the absorbance maximum at a wavelength of 506 nm. The scan was performed with a 0.1 mg mL^{-1} solution of Sudan III in 96% (V/V) ethanol using a Tecan 20M multiplate spectrophotometer.

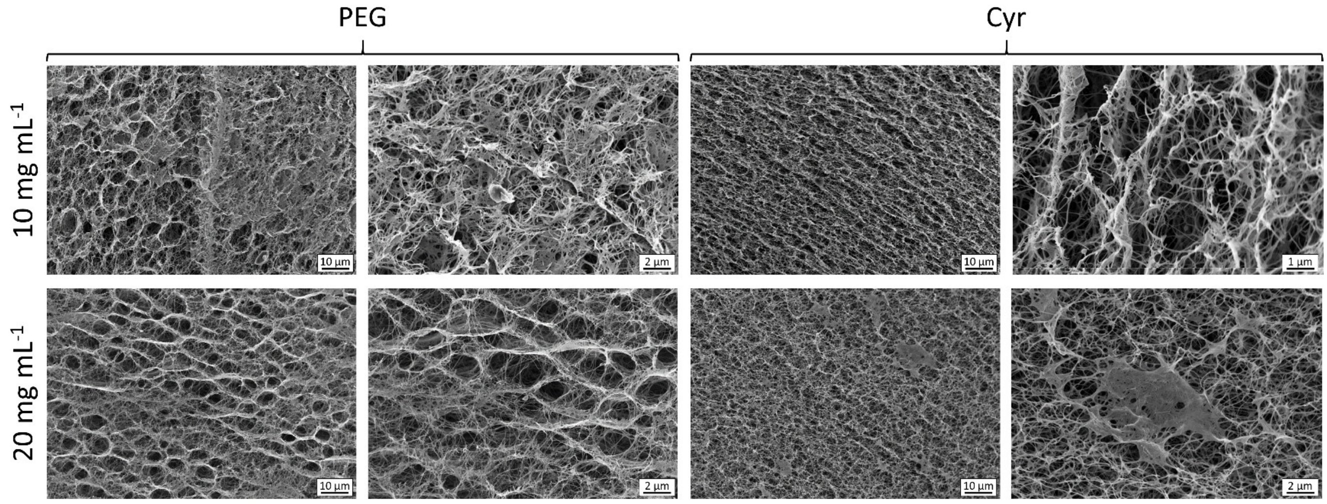

Figure S2. Morphology of the PLGA phase within BNC depicted by scanning electron microscopy (1,000 to 10,000 x magnification) of the cross section of the fleeces after precipitation of 10 and 20 mg mL^{-1} PLGA from the solvents PEG and Cyr and subsequent freeze-drying.

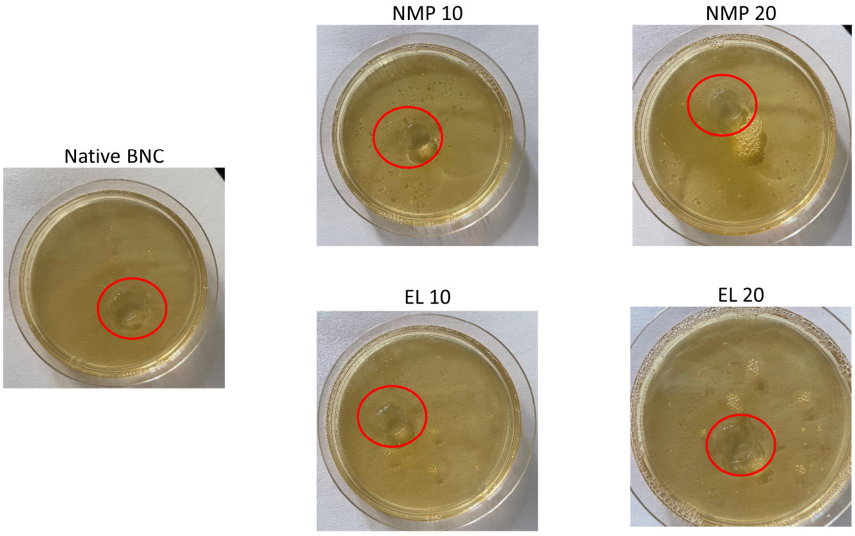

Figure S3. Pictures of gelatin gels after 48 h of incubation in the fluid release experiments. The visibly swollen contact areas to the BNC fleeces are highlighted.

Table S1. Gradient of mobile phase for CBD HPLC analysis.

Time [min]	Percentage of mobile phase A [%]	Percentage of mobile phase B [%]
0	100	0
22	0	100
24	100	0
25	100	0

Table S2. Mathematical modelling of cumulative CBD release profiles applying Ritger and Peppas' semi-empirical power-law.

Formulation	Power-Law Equation	Diffusion exponent *n*	Linear regression R^2
Propylene glycol	y = 0.4481x + 1.0433	0.45	0.972
NMP 10	y = 0.7471x + 0.2726	0.75	0.999
NMP 20	y = 0.7861x + 0.2086	0.79	0.999
EL 10	y = 1.0245x - 0.1895	1.02	0.999
EL 20	y = 0.9548x – 0.1315	0.95	0.999

Table S3. Mathematical modelling of cumulative CBD release profiles applying first-order kinetics, zero-order kinetics and the Higuchi square root equation.

Formulation	First-order R^2	Zero-order R^2	Higuchi R^2
Propylene glycol	0.536	0.813	0.952
NMP 10	0.883	0.990	0.996
NMP 20	0.897	0.994	0.992
EL 10	0.967	0.999	0.998
EL 20	0.883	0.998	0.985

Table S4. Mathematical modelling of cumulative AKBA release profiles applying Ritger and Peppas' semi-empirical power-law.

Formulation	Power-Law Equation	Diffusion exponent *n*	Linear regression R^2
EL 10	y = 0.9274x + 0.8382	0.93	0.995
EL 20	y = 0.9876x + 0.6505	0.99	0.995

References

1. Karl, B.; Alkhatib, Y.; Beekmann, U.; Bellmann, T.; Blume, G.; Steiniger, F.; Thamm, J.; Werz, O.; Kralisch, D.; Fischer, D., Development and characterization of bacterial nanocellulose loaded with Boswellia serrata extract containing nanoemulsions as natural dressing for skin diseases. *Int. J. Pharm.* **2020,** *587*, 119635. doi: https://doi.org/10.1016/j.ijpharm.2020.119635
2. ICH guideline Q2 (R1), Validation of analytical procedures: text and methodology International Council for Harmonisation of Technical Requirements for Pharmaceuticals for Human Use: 2005; Vol. 4, pp 1-17.
3. Moritz, S.; Wiegand, C.; Wesarg, F.; Hessler, N.; Müller, F. A.; Kralisch, D.; Hipler, U. C.; Fischer, D., Active wound dressings based on bacterial nanocellulose as drug delivery system for octenidine. *Int. J. Pharm.* **2014,** *471* (1-2), 45-55. doi: https://doi.org/10.1016/j.ijpharm.2014.04.062
4. Bellmann, T.; Luber, R.; Kischio, L.; Karl, B.; Pötzinger, Y.; Beekmann, U.; Kralisch, D.; Wiegand, C.; Fischer, D., Bacterial nanocellulose patches as a carrier for hydrating formulations to improve the topical treatment of nail diseases. *I Int. J. Pharm.* **2022,** *628*, 122267. doi: https://doi.org/10.1016/j.ijpharm.2022.122267

Disclaimer/Publisher's Note: The statements, opinions and data contained in all publications are solely those of the individual author(s) and contributor(s) and not of MDPI and/or the editor(s). MDPI and/or the editor(s) disclaim responsibility for any injury to people or property resulting from any ideas, methods, instructions or products referred to in the content.
